# Revealing the role of the *AGO4* gene against rice *hoja blanca* virus: from transformation to protein structure

**DOI:** 10.3389/fpls.2025.1517321

**Published:** 2025-03-28

**Authors:** Johan Ñañez, Sandra Valdes, Maribel Cruz Gallego, Maria Camila Rebolledo, Mathias Lorieux, Maria Fernanda Alvarez, Paul Chavarriaga

**Affiliations:** ^1^ Department of Biotechnology, Gene Editing Platform, Alliance Bioversity International - Centro Internacional de Agricultura Tropical (CIAT), Palmira, Colombia; ^2^ FLAR, Fondo Latinoamericano para Arroz de Riego, Palmira, Colombia; ^3^ OMICAS, Pontificia Universidad Javeriana, Cali, Colombia; ^4^ Plant Diversity, Adaptation and Development (DIADE), University of Montpellier, Center for International Cooperation in Agricultural Research for Development (CIRAD), Institut de Recherche pour le Développement (IRD), Montpellier, France

**Keywords:** *Oryza sativa*, *Tenuivirus*, Alphafold2, argonaute protein, CRISPR/Cas9

## Abstract

Rice *hoja blanca* virus (RHBV), transmitted by the insect vector *Tagosodes orizicolus*, poses a significant threat to rice cultivation. Here, we use CRISPR/Cas9 technology to produce specific mutations in the *AGO4* gene of *Oryza sativa*, using the Fedearroz 2000 variety, with the aim of elucidating the participation of the gene in resistance to RHBV. We obtained 14 edited plants that presented with deletions of one, two, and three nucleotides in the sequence of exon 23 of the *AGO4* gene. Phenotypic evaluations showed an increase in susceptibility to RHBV in the edited lines. We identified the presence of RHBV in the leaf tissue of infected plants by amplifying the nucleoprotein, *NS3*, and *NS4* genes of the virus. Using RT-qPCR, we analyzed the expression patterns of the *AGO4* gene, showing that in the edited lines, the expression profiles are similar to the susceptible control. Furthermore, modeling of the tertiary structure of the AGO4 protein and its mutant variant demonstrated changes in the PIWI domain and the presence of the DDH catalytic triad, confirming its role in mediating resistance to RHBV. Our study reveals the functional importance of the rice *AGO4* gene in RHBV resistance.

## 1 Introduction

Rice (*Oryza sativa*) is one of the main components of food security in developing countries in Africa and South America (Sasaya et al., 2014; Sen et al., 2020). It is consumed by more than half of the world’s population. Its cultivation is of great importance to ensure the sustenance of large populations and, therefore, contributes to the economy of various countries. However, there are different biotic factors that can affect rice crops. Diseases caused by viral pathogens alter plant growth, reducing grain production and quality.

In tropical America, the rice *hoja blanca* virus (RHBV) is a single-stranded RNA (mcRNA) virus that belongs to the *Tenuivirus* genus. This group includes the rice streak virus (RSV), the maize streak virus (MSV), and the European wheat streak mosaic virus (EWSMV), characterized by infecting plants of agricultural interest by being transmitted by insects that constitute their main transmission vectors (Malathi and Mangrauthia, 2013; Scheel et al., 2016; Martin et al., 2020; Feng et al., 2021). Its genome is negative-sense or ambisense, composed of four to six mcRNA segments, where each segment encodes proteins essential for its subsistence (Nguyen and Haenni, 2003). RNA1 encodes the RNA-dependent RNA polymerase. The non-structural protein *NS2* is encoded in RNA2 together with the membrane glycoprotein. The *NS3* gene responsible for silencing the host’s viral suppression mechanisms is located on RNA3. Finally, the RNA-associated nucleoprotein (*NCP*) and the non-structural protein *NS4* are encoded on RNA4 (Morales and Jennings, 2010; Jimenez et al., 2018). Some conspicuous symptoms are chlorotic straight lines that coalesce, turning green leaves white or yellowish tones. Early infections stunt young plants, generate necrosis, and even result in the death of plants. Late infections, before panicle formation, reduce seed production and affect grain quality (Romero et al., 2014; Bolanos et al., 2017; Cruz-Gallego et al., 2018). This disease can generate significant losses, ranging from 25% to 100%, depending on the susceptibility of the variety planted. RHBV is transmitted by *Tagosodes orizicolus* Muir (Hemiptera: Delphacidae), an insect that is used as a virus vector. When feeding, *T. orizicolus* sucks nutrients present in the leaves of rice plants and simultaneously secretes a substance produced in the salivary glands, one of the sites where the virus grows in infected insects for transmission (Cruz-Gallego et al., 2018).

Although there are varieties of *O. sativa* resistant to RHBV, through RNA interference (RNAi), plants defend themselves against infection by eliminating the viral RNA (Muthamilarasan and Prasad, 2013). The silencing of the viral RNA (siRNA) is one of the main resistance mechanisms in plants (Sasaya et al., 2014; Zhang et al., 2015). When a plant is infected, the dicer (DCL) proteins recognize the dsRNA and produce siRNA from the genetic material of the virus, subsequently, the siRNA is recruited by the RNA silencing complex (RISC) associated with argonaute proteins (AGO), which by complementarity recognize the viral mRNA and generate silencing (Muthamilarasan and Prasad, 2013; Deng et al., 2022). AGO encompass an entire family highly conserved in eukaryotic organisms, responsible for binding to miRNA or sRNA fragments, forming complexes for transcriptional gene silencing (TGS) and post-transcriptional gene silencing (PTGS) for the regulation of plant development, regulation of expression genetics, and immunity or resistance to viral pathogens (Mallory and Vaucheret, 2010; Zhang et al., 2015).

Viruses have developed strategies to infect and thrive inside plants and insects by evading degradation. Viral suppressors of RNA silencing (VSRSs) are a strategy developed to counteract host defense mechanisms. VSRSs aim to inhibit or decrease the effectiveness of RNAi, allowing dsRNA to replicate normally. To accomplish this task, some VSRSs sequester siRNA produced by the action of DCL proteins, preventing the RISC complex from being guided. Others interfere with the activity of Dicer nucleases, blocking the production of siRNA. Finally, there are VSRSs that act directly on the correct functioning of AGO proteins (Moissiard and Voinnet, 2006; Xiong et al., 2009; Kormelink et al., 2011; Zhao et al., 2021). In *Tenuiviruses*, the *NS3* gene can bind to both dsRNA and siRNA, which blocks its incorporation into the RISC complex, thus preventing it from being targeted for degradation (Shen et al., 2010; Yang et al., 2011).

The study by Tu et al. (2023) revealed that the AGO5 protein in *Nicotiana benthamiana* (NbAGO5) plays a crucial role in antiviral defense by binding viral small interfering RNAs (vsiRNAs) and activating RNA silencing to inhibit various viruses, including Bamboo mosaic virus (BaMV), Potato virus X (PVX), tobacco mosaic virus (TMV), and a mutant variant of cucumber mosaic virus (CMV). However, some viruses, such as wild-type CMV and turnip mosaic virus (TuMV), counteract this defense by degrading NbAGO5 through the 26S proteasome and autophagy pathways or interfering with its ability to bind vsiRNAs. This study highlights the complexity of the interactions between NbAGO5-mediated defense mechanisms and viral counter-defense strategies.

Argonaute (AGO) proteins, key components of the RISC complex, play a central role in post-transcriptional gene silencing (PTGS) and transcriptional gene silencing (TGS). AGO proteins in the RISC complex recognize and bind, by complementarity, to viral RNA thanks to the guide siRNA; where, by endonuclease-like activity, they generate cuts or sequester and inhibit the translation of the viral material, ending the degradation of the virus (Song et al., 2004; Ender and Meister, 2010; Liang et al., 2023).

Phylogenetic analyses in plant organisms group the different AGO proteins into three main clades: AGO1/5/10 (clade 1), AGO2/3/7 (clade 2), and AGO4/6/8/9 (clade 3) (Baumberger and Baulcombe, 2005; Zhou et al., 2016). Their functions vary depending on the developmental stage of the plants and their interactions with the environment, depending on the type of RNA to which they are associated. Their PAZ, MID, and PIWI domains structure and differentiate the functional areas of the proteins. PAZ and MID contain a specific binding site for the 3’ end of the sRNA, while the structure of the PIWI domain shares similarity with a bacterial RNAase H with the ability to cleave RNA or DNA chains (Ender and Meister, 2010; Zhang et al., 2015; Liang et al., 2023).

An investigation conducted by Romero et al. (2014) focused on identifying the quantitative trait loci (QTLs) that make rice varieties resistant to RHBV, finding an important QTL for resistance to RHBV in a region of the short arm of chromosome 4. Later, Silva et al. (2023) identified the same QTL in resistant varieties, such as Fedearroz 2000, naming it qHBV4.1. An analysis of the genes present in the qHBV4.1 QTL region allowed the identification of the *AGO4* gene as the possible viral defense agent against RHBV in resistant varieties.

Here, we present the relationship between the *AGO4* gene and resistance to RHBV in the Fedearroz 2000 variety by obtaining plants edited with the CRISPR/Cas9 system. We identified that by editing exon 23 of the *AGO4* gene and exposing the edited plants to the virus, an increase in susceptibility was evident as the incidence of RHBV increased. We report the relative expression levels due to the editing of the *AGO4* gene and 3D modeling of the protein in response to increased susceptibility to RHBV.

## 2 Materials and methods

### 2.1 Plant materials

We use the resistant rice variety Fedearroz 2000 (FD2000) for the transformation. The Bluebonnet 50 (BB50) variety was used as a susceptible control, and the Colombia 1 (Col1) variety as an intermediate control. Plants were grown in a biosafety greenhouse.

### 2.2 Target sequences selection

**Figure 1:**
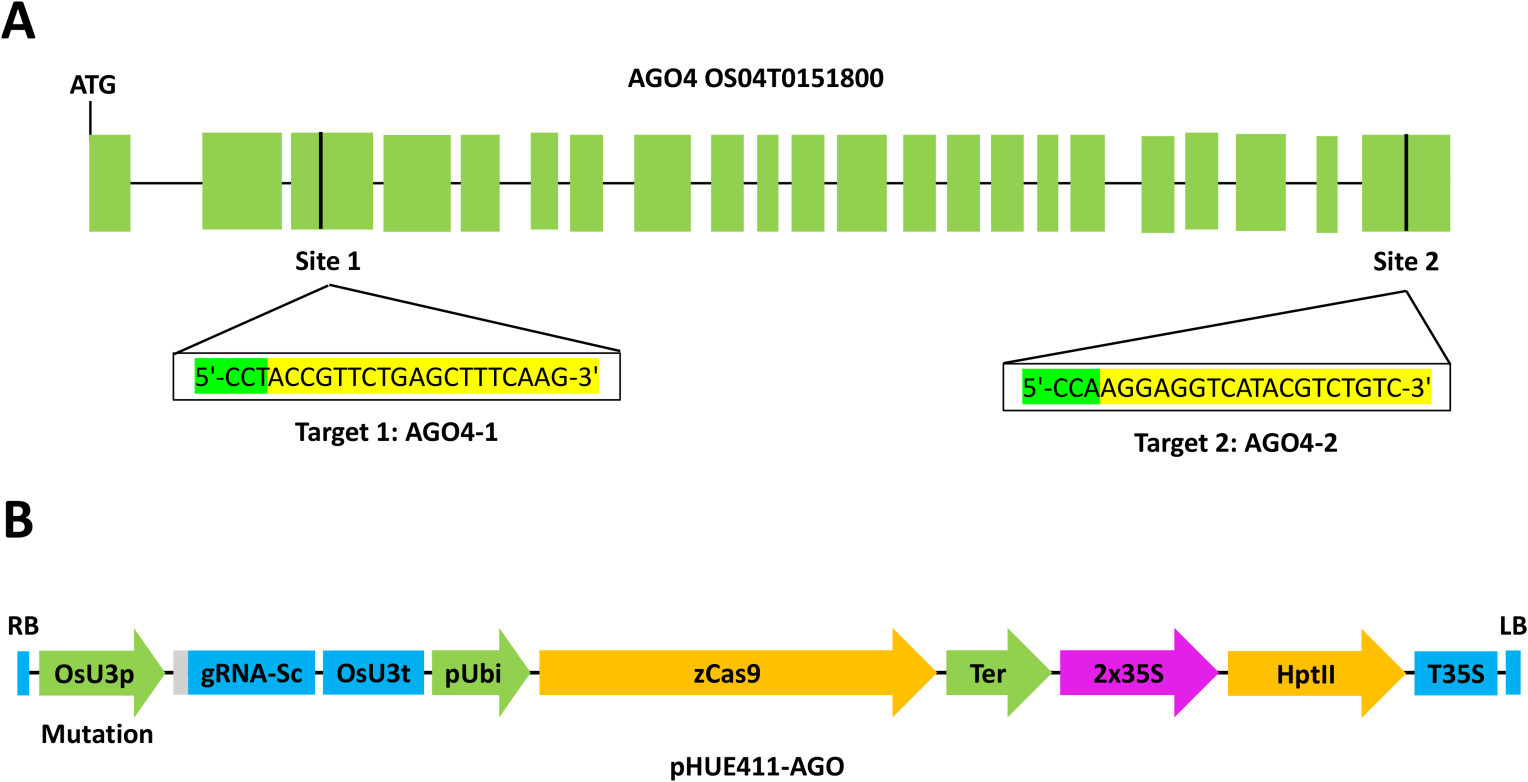
Transformation of FD2000 with the CRISPR/Cas9 system. **(A)** Graphic scheme of the AGO4 gene (OS04T0151800). Target 1 is marked in the third exon and target 2 in exon 23. **(B)** Graphic representation of the transformation plasmid pHUE411-AGO.

In order to validate the effect of the *AGO4* gene on *hoja blanca* virus resistance, two targets were designed on the *OS04T0151800* gene. The first target (AGO4-1) was engineered in the third exon and the second target was engineered in exon 23 (**Figure 1**). Both targets were chosen through the CRISPR-P 2.0 (http://crispr.hzau.edu.cn/CRISPR2/) platform, which allowed us to identify potential off-targets for those regions (**Supplementary Table S1**). The sgRNA-Cas9 plant expression vector was purchased from Addgene (www.addgene.org, MA, US). The vector was constructed by inserting synthesized oligos into the BsaI site of the vector pHUE411, which contains a codon-optimized Cas 9 driven by a Ubiquitin promoter and a sgRNA scaffold directed by rice U3 promoter.

### 2.3 Rice transformation

The *Agrobacterium tumefaciens* strain EHA105 was used to transform immature embryos of FD2000. The genetic transformation was carried out as described by Liu et al. (2020). Once the plants were regenerated, they were transferred to a biosafety greenhouse. One week after the establishment of the lines, DNA extractions were performed to evaluate the presence of *hygromycin* and *ubiquitin* promoter to identify positive transgenic plants. Non-transgenic plants were obtained for target one (AGO4-1), and 20 lines were obtained for target two (AGO4-2). In total, 50 seeds of each line were cultivated under greenhouse conditions with the objective of obtaining DNA-free lines. All of them were evaluated by PCR to determine the presence of *hygromycin* (HPT) and Cas9 (**Table 1**). Some of the lines were sequenced (**Table 2**), obtaining deletions of one, two, and three nucleotides in the region of interest.

**Table 1:** AGO4-2 lines generated with the CRISPR/Cas9 system.

Línes ago4-2 T0	Molecular ID ago4-2-T0	Lines ago4-2-T1	Lines ago4-2-T1
Os 2016-33-52	AGO4-2P4	Os 2016-33-52-T1-plant 2	AGO4-2P4-1
		Os 2016-33-52-T1-plant 15	AGO4-2P4-2
		Os 2016-33-52-T1-plant 21	AGO4-2P4-3
Os 2016-33-36	AGO4-2P22	Os 2016-33-36-1-plant 13	AGO4-2P22-1
		Os 2016-33-36-1-plant 38	AGO4-2P22-2
Os 2016-58-5	AGO4-2P38	Os 2016-58-5-1-plant 31	AGO4-2P38-1
		Os 2016-58-5-1-plant 40	AGO4-2P38-2
		Os 2016-58-5-1-plant 41	AGO4-2P38-3
		Os 2016-58-5-1-plant 42	AGO4-2P38-4
Os 2016-58-3	AGO4-2P15	Os 2016-58-3-1-plant 17	AGO4-2P15-1
		Os 2016-58-3-1-plant 25	AGO4-2P15-2
		Os 2016-58-3-1-plant 26	AGO4-2P15-3
		Os 2016-58-3-1-plant 32	AGO4-2P15-4
Os 2016-68-11	AGO4-2P33	Os 2016-68-11-1-plant 25	AGO4-2P33-1

**Table 2:** Sequence of the WT and the edited lines AGO4-2.

Molecular ID	Sequence
WT	GTCGGACGCGTCGTCCAGCCAAGGAGGTCATACGTCTGTCGGAAGTGTACCGGTGCCTGA
AGO4-2P4-1	GTCGGACGCGTCGTCCAGCCAAGG-GGTCATACGTCTGTCGGAAGTGTACCGGTGCCTGA
AGO4-2P4-2	GTCGGACGCGTCGTCCAGCCAAGG-GGTCATACGTCTGTCGGAAGTGTACCGGTGCCTGA
AGO4-2P4-3	GTCGGACGCGTCGTCCAGCCAAGG-GGTCATACGTCTGTCGGAAGTGTACCGGTGCCTGA
AGO4-2P22-1	GTCGGACGCGTCGTCCAGCCAAG–GGTCATACGTCTGTCGGAAGTGTACCGGTGCCTGA
AGO4-2P22-2	GTCGGACGCGTCGTCCAGCCAAG–GGTCATACGTCTGTCGGAAGTGTACCGGTGCCTGA
AGO4-2P38-2	GTCGGACGCGTCGTCCAGCCAAGG-GGTCATACGTCTGTCGGAAGTGTACCGGTGCCTGA
AGO4-2P38-4	GTCGGACGCGTCGTCCAGCCAAGG-GGTCATACGTCTGTCGGAAGTGTACCGGTGCCTGA
AGO4-2P15-2	GTCGGACGCGTCGTCCAGCCAA—GGTCATACGTCTGTCGGAAGTGTACCGGTGCCTGA
AGO4-2P33-1	GTCGGACGCGTCGTCCAGCCAAGG-GGTCATACGTCTGTCGGAAGTGTACCGGTGCCTGA

### 2.4 Evaluation of RHBV incidence in the AGO4-2 lines

**Figure 2:**
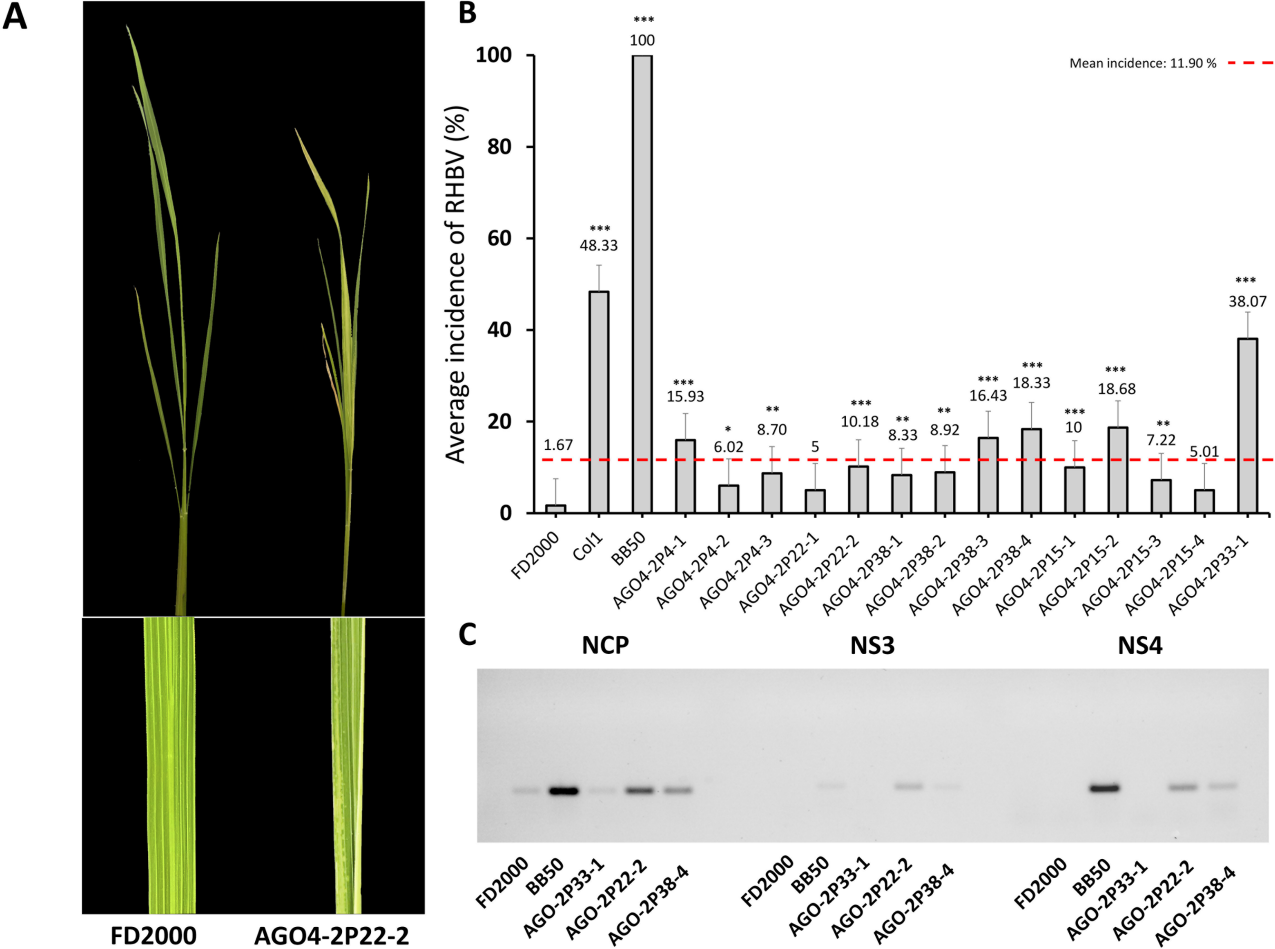
Phenotypic and genotypic characterization of RHBV in control and edited lines. **(A)** Symptoms of RHBV. FD2000 resistant to RHBV does not present chloritic stains. The edited line AGO4-2P22-2 presents a partial appearance of chlorotic spots. **(B)** Average incidence of RHBV in the edited lines AGO4-2, FD2000, BB550, and Col1. **(C)** Amplification of the RHBV NP, *NS3*, and *NS4* genes in leaves of FD200, BB50, and AGO4-2 edited plants (2P33-1, 2P22-2, and 2P38-4). Statistical significance *P < 0.05, **P < 0.01, ***P < 0.001. The red dashed line indicates the global average incidence of the AGO4-2 lines, 95% confidence interval: 11.90%.

The incidence of RHBV in the edited AGO4-2 lines was estimated by sowing the second-generation (T2) plants in plastic trays with 14 rows. Each row contained 20 plants selected for the absence of HPT and Cas9. Each tray contained a row for resistant, intermediate, and susceptible controls to RHBV: FD2000 (resistant), Col1 (intermediate), and BB50 (susceptible). Furthermore, 18 days after sowing, the trays were placed into mesh cages (one tray per cage) and were infested by mass release of the insect vector *T. orizicolus*, with an average of four nymphs per plant. For a period of 3 days, the nymphs fed on plant tissue, and then the plants were disinfested. The evaluation of the incidence was carried out after 35–40 days post infestation, counting the plants per row that showed the RHB symptoms (**Figure 2**). The presence of RHBV was evaluated in the edited plants through the amplification of the NP, *NS3*, and *NS4* genes of the virus (**Figure 2C**).

### 2.5 Viral infection of AGO4-2 lines with RHBV

Before planting the experimental lines, insects were tested to ascertain if they were virulent or not, using the susceptible variety BB50. One insect per plant was isolated using acetate tubes (**Supplementary Figure S1**). After 18 to 20 days, if the plants showed symptoms of RHBV, this meant that the insect was a vector, and if the plants showed no symptoms, the insect was a non-vector. The selected insects were used in subsequent infestations.

The AGO4-2 lines, the WT (F2000), and the susceptible control line (BB50) were grown under greenhouse biosafety conditions, with 1.5 grams of seed per line (40-60 seeds) sown. Furthermore, 12 days after seeding, nine individual plants were left, and three treatments (three plants per treatment) were managed per line: plants without insects (treatment 1), plants with non-vector insects (treatment 2) and plants with virus vector insects (treatment 3). Finally, on day 18, the lines were infested using two insects of the same genus (two males or two females) per plant. To individualize each plant, acetate tubes were covered by a fabric net (**Supplementary Figure S1**). After 3 days, the plants were disinfested by vacuuming up the insects. On the fourth (120 h) and sixth (168 h) days after infestation, young leaf tissue was collected in liquid nitrogen and stored at −80°C.

### 2.6 RNA extraction and cDNA synthesis

Total RNA from the transgenic and WT lines was extracted using TRIzol™ Reagent (Invitrogen). The purity and concentration of the RNA obtained were quantified using Nanodrop One (Thermo Fisher Scientific). The integrity and quality of the RNA were evaluated by electrophoresis in a 1.5% agarose gel. RNA purification and cDNA synthesis were performed using the QuantiTect Reverse Transcription Kit (QIAGEN), following the manufacturer’s instructions.

### 2.7 RT-qPCR

The real-time PCR assays were performed using QuantStudio 5 equipment (Thermo Fisher Scientific) with the PowerUp™ SYBR™ Green Master Mix (Applied Biosystems). The amplification program used was: 95°C for 2 min, 40 cycles of 95°C for 5 s, 58°C for 30 s, and a melting curve. The data were normalized according to the rice genes: elongation factor 1-alpha 1 (*eEF-1a*) and glyceraldehyde-3-phosphate dehydrogenase (*GAPDH*). Relative expression levels were calculated using the Delta-Delta Ct $\left(2^{–\Delta \Delta Ct}\right)$ comparative relative quantification method. The amplification efficiency was measured by evaluating the primers using the standard curve, maintaining an efficiency close to 100%. There were three biological replicates for each treatment and two technical replicates. The trials were repeated at two different times without significant variation in the results. For statistical analysis, the one-way ANOVA test was used to identify significant expression differences. The primers used in this study can be found in the **Supplementary Material** (**Supplementary Table S2**).

### 2.8 Alignment and homology modeling of protein structures

The domains of the AGO4 protein were determined using the InterProScan tool (https://www.ebi.ac.uk/interpro/) and the alignment of the protein sequences was carried out in the Clustal Omega tool (https://www.ebi.ac.uk/jdispatcher/msa/clustalo).

The tertiary structure was modeled using the SWISS-MODEL homology modeling platform (https://swissmodel.expasy.org). The amino acid sequences of the FD2000 and mutant lines were entered. The Q9SDG8.1.A template was selected based on the GMQE, QMEAN4 values, ​​and the identity percentage as the optimal template to model the AGO4 protein. The mutant versions were obtained using the Alphafold2 method. The Swiss-PdbViewer and ChimeraX programs were used for modeling processing and analysis.

## 3 Results

### 3.1 Rice transformation

In the transformation process of the resistant variety FD2000, two positions were targeted (**Figure 1A**). We obtained T0 plants for target 2 (AGO4-2), but for the target at site 1 (AGO4-1) no transgenic plants were produced. Five AGO4-2 transgenic lines (2P4, 2P22, 2P38, 2P15, and 2P33) were selected by PCR for the presence of the HPT and *ubiquitin* transgenes. They were segregated until a T1 plant free of HPT and Cas9 was produced from each minimal line, obtaining 14 edited plants free of T-DNA (**Table 1**). T0 and T1 plants were sequenced to determine the edition generated. On the AGO4-2 lines, 2P4, 2P38, and 2P33 deletion of 1 nt occurred. In line 2P22, there was a deletion of 2 nt and in the 2P15 line, 3 nt were deleted within the target site (**Table 2**).

### 3.2 Susceptibility to RHBV increased in the edited AGO4-2 lines

To evaluate phenotypically the resistance to RHBV in the edited lines in the T2 generation with one, two, and three nucleotide deletion mutations (**Table 2**), susceptible control, intermediate control, and WT plants were subjected to viral stress through infestation with *T. orizicolus*, the virus vector. The average incidence was determined according to the number of plants per line that showed RHBV symptoms (**Figure 2A**). There was an increase in the incidence of RHBV of 38.07% in the AGO4-2P33-1 line, followed by the AGO4-2P15-2 and AGO4-2P38-4 lines with an increase of 18.68% and 18.33%, respectively. The AGO4-2P38-2, AGO4-2P4-1, and AGO4-2P22-2 lines presented an increase greater than 10% (**Figure 2B**). The WT presented an average virus incidence of 1.67%. In contrast, the susceptible control BB50 obtained a 100% incidence. Additionally, the presence of the virus was identified by amplifying the NP genes and the non-structural proteins *NS3* and *NS4* in the leaf tissue of the infected lines. There was evidence that, in the WT line, the presence of the virus post-infection was low in terms of NP amplification and null in the non-structural proteins. In contrast, the amplification of the virus genes was significant in the susceptible control BB50. As for the edited plants, the AGO4-2 lines 2P22-2 and 2P38-4 presented amplification of the three virus genes, a pattern similar to the susceptible control BB50. Line 2P33-1 only showed amplification of NP, a protein directly associated with dsRNA (**Figure 2C**).

### 3.3 Expression levels of the AGO4 gene in the WT, BB50, and AGO4-2 lines

**Figure 3:**
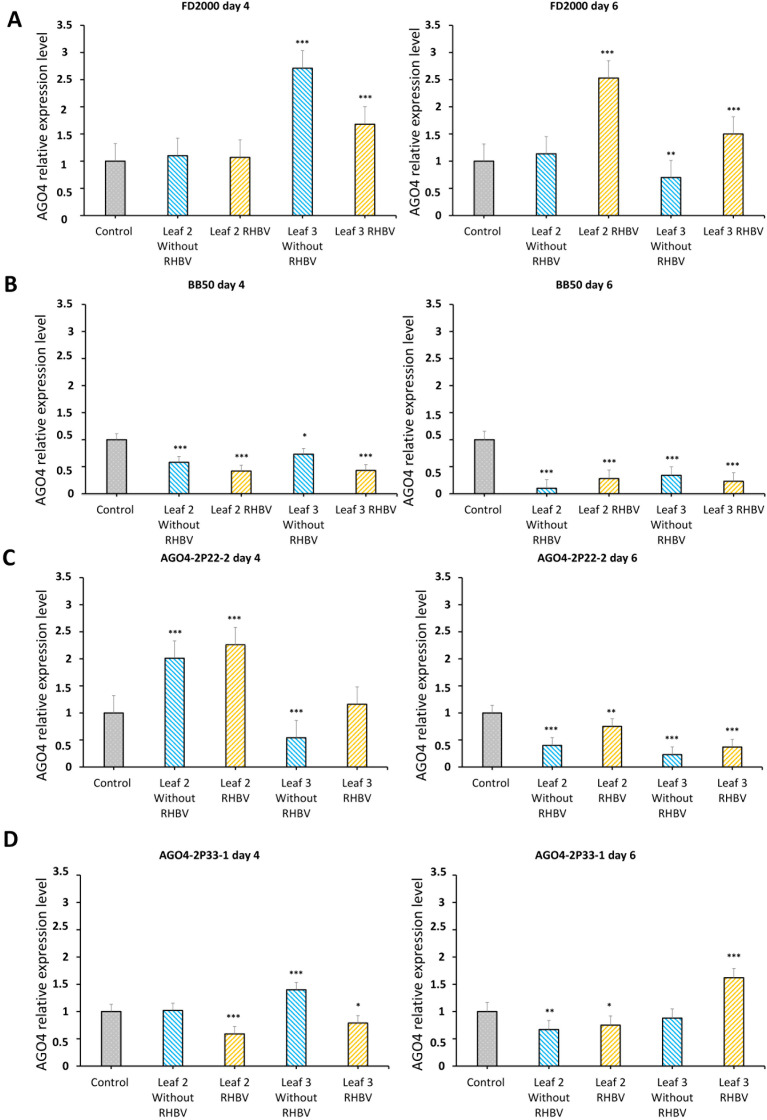
Expression profile of the AGO4 gene. **(A)** AGO4 gene expression pattern in FD2000 4 and 6 days after viral (RHBV) and mechanical (without RHBV) stress. **(B)** AGO4 gene expression pattern in BB50 4 and 6 days after viral (RHBV) and mechanical stress (without RHBV). **(C)** Expression pattern of the AGO4 gene in the AGO4-2P22-2 line 4 and 6 days after viral (RHBV) and mechanical (without RHBV) stress. **(D)** Expression pattern of the AGO4 gene in the AGO4-2P33-1 line 4 and 6 days after viral (RHBV) and mechanical (without RHBV) stress. Statistical significance *P < 0.05, **P < 0.01, ***P < 0.001.

The expression pattern of the AGO4 gene was obtained in the tissue of young leaves 18 days after sowing (leaf two and leaf three), during two specific times on the fourth and sixth day post-infestation (**Supplementary Figure S2**). The expression of the AGO4 gene was measured in the WT line, the AGO4-2 edited lines 2P22-2 and 2P33-1, and the susceptible control line BB50. The plants were infested with insect vectors (RHBV) and non-infected insects (without RHBV) in order to determine if the expression of the gene was directly related to the viral stress caused by the presence of RHBV or if it was due to the mechanical stress produced by *T. orizicolus* insects when feeding and ovipositing on the veins of the leaves. The WT line resistant to RHBV showed a similar expression level to the control (non-infested plants) for both treatments on the fourth day in leaf two, while leaf three presented a significant increase in expression levels for both mechanical damage (without RHBV) and viral stress (RHBV). Furthermore, on the sixth day, only plants infected with the virus showed overexpression of AGO4 in both tissues and this decreased in plants that were not infected (**Figure 3A**). In contrast, the susceptible line BB50 presented low levels of relative expression of the AGO4 gene for both treatments in all tissues (**Figure 3B**).

The AGO4-2P22-2 line showed overexpression of the gene for both treatments compared to the control on the fourth day on the second leaf, while on leaf three, there was a decrease in expression due to mechanical stress (without RHBV). In the viral stress treatment, gene expression did not have significant changes compared to the control. Furthermore, by the sixth day, the expression of the line decreased significantly for both tissues and treatments (without RHBV and with RHBV), an expression profile similar to that of the susceptible control line BB50 (**Figure 3C**). The AGO4-2P33-1 line on the fourth day showed a decrease in expression in plants with viral stress (RHBV) in both tissues. Leaf two without RHBV did not show significant changes in relation to the control, while in leaf number three without RHBV, the expression increased. By the sixth day, the line presented a decrease in expression levels for both treatments in leaf two. For leaf three, the treatment without RHBV did not generate significant changes in the relative expression of the AGO4 gene, but the plants infected with RHBV had an overexpression (**Figure 3D**).

### 3.4 AGO4 protein

**Figure 4:**
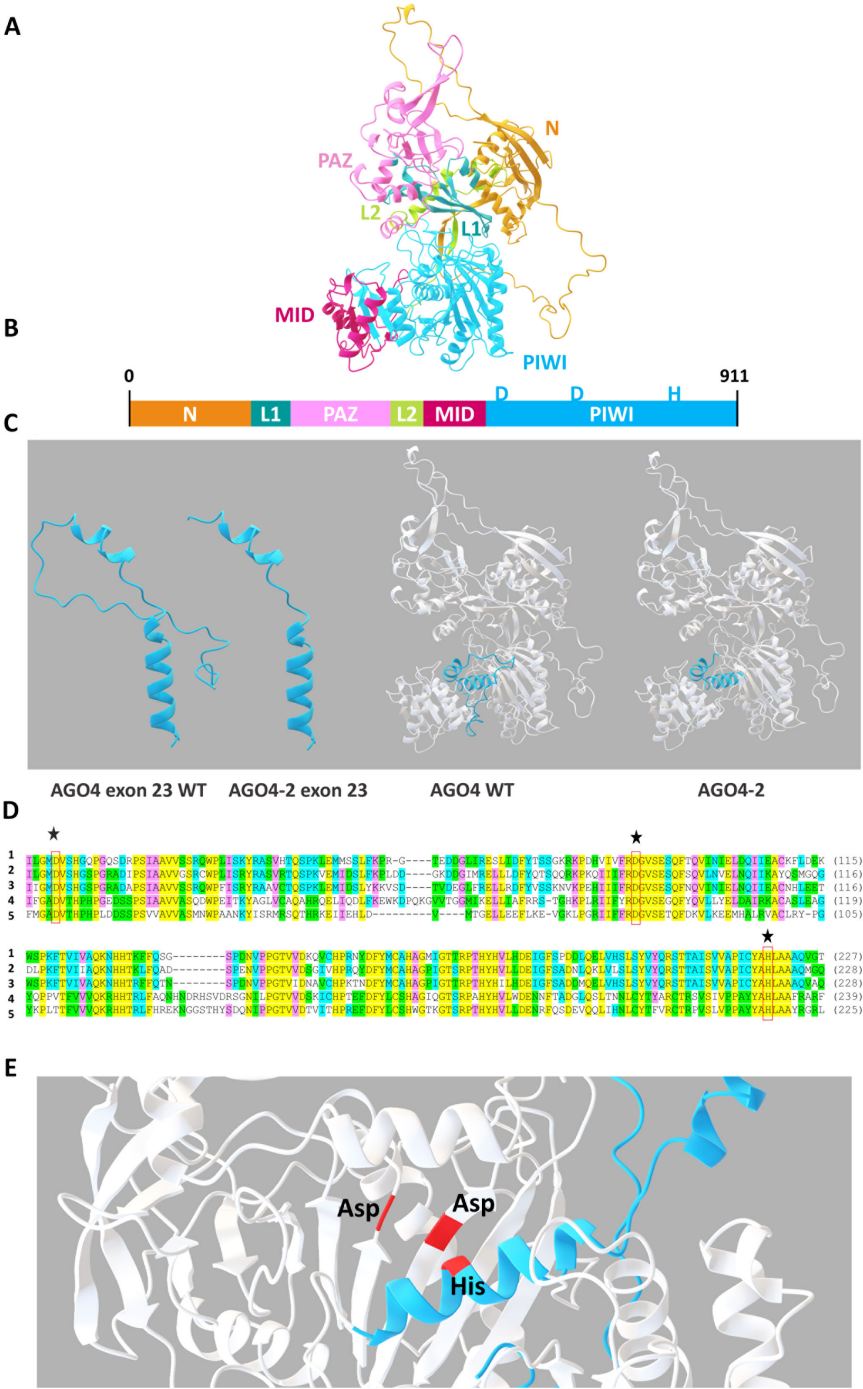
Homology modeling of the AGO4 protein. **(A)** Tertiary structure of the AGO4 protein. **(B)** Graphical schematic of the N-terminal, PAZ, MID, and PIWI domains and the L1 and L2 linker segments. **(C)** Multiple alignments of the amino acid sequences of the argonaute proteins of the species (1) *Oryza sativa* AGO4, (2) *Arabidopsis thaliana* AGO1, (3) *Zea mays* AGO6, (4) *Solanum lycopersicum* AGO4, and (5) *Triticum aestivum* AGO with previous registration of RNA silencing activity. Stars indicate the position of residues of the DDH catalytic triad. **(D)** Tertiary structure of the AGO4 protein highlighting the section corresponding to exon 23 and its mutant version. **(E)** Location of the DDH catalytic triad.

To understand the function of the AGO4 gene in relation to RHBV-resistance in the WT, the tertiary structure of the protein and its mutant version were modeled (**Figure 4**). The presence of the N-terminal (N), two connecting bridges (L1 and L2), and three functional domains (PAZ, MID, and PIWI) that make up the structure of the protein was determined (**Figures 4A, B**). Multiple alignment was performed with the amino acid sequences of the PIWI catalytic domain of different argonaute proteins from the species *Arabidopsis thaliana* (AGO1), *Zea mays* (AGO6), *Solanum lycopersicum* (AGO4), and *Triticum aestivum* (AGO7), with previous registration of silencing activity of RNA, compared to the WT sequence. We found that the PIWI domain had a high degree of similarity between the AGO proteins with siRNA function from different plant species. The presence of the DDH catalytic triad (Asp-Asp-His) was detected in the WT (**Figure 4C**). The DDH residues form the active RNA cleavage site. When obtaining the 3D structure of the AGO4 protein, the residues were located close to each other within the catalytic site (**Figure 4E**). Modeling the editing in exon 23 compared to the WT, they were compared separately, and we located the editing in the protein, demonstrating the change in the tertiary structure by losing part of the PIWI domain and the C-terminal due to the presence of a stop codon at the end of the sequence due to the generated edit (**Figure 4D**).

## 4 Discussion

Argonaute proteins are highly conserved in eukaryotic organisms. They play an important role in regulating the expression of endogenous genes or controlling viral infections through transcriptional gene silencing and post-transcriptional gene silencing (Mallory and Vaucheret, 2010). When we mutated the AGO4 gene of *O. sativa* in the RHBV-resistant variety FD2000, we obtained two scenarios. Editing in target 1 (AGO4-1) of exon 3 produced a knockout stop codon, eliminating the function of the gene. The seeds obtained from the T0 plants of the AGO4-1 lines did not germinate. It has been recorded that AGO4 in different plant species, such as *Triticum aestivum L*., plays a key role in activating the expression of genes that encode enzymes required for seed germination that decompose endosperm starch into sugars for the nutrition of embryos (Meng et al., 2013). The editing in target 2 of exon 23 similarly generated a stop codon, the result of the deletion of one to two nucleotides, affecting only the last stretch of the gene sequence. The seeds of the T0 plants managed to germinate since the editing produced a knockdown of the gene. This is reflected in the decrease in the number of germinated seeds compared to the number of seeds sown. The seedlings that managed to germinate grew at a slow rate compared to the WT, evidencing in the first instance a malfunction of the gene in the early stages of development due to the mutation generated.

As a result of the resistance presented by FD2000, the average incidence of RHBV was 1.67%. We demonstrated how, by mutating the AGO4 gene and decreasing its activity, susceptibility increased between 10% and 38.07%, reflected in the appearance of RHBV symptoms in the leaves of the edited plants and the amplification of the NP, *NS3*, and *NS4* genes of the virus in infected plants. In *Tenuiviruses*, the NP gene encodes a structural protein associated with the viral RNA. It fulfills the function of stability of the genetic material and resistance to degradation by RNase and is closely related to the replication of the virus in the host organism. The *NS3* gene encodes a non-structural protein with the function of suppressing the RNA silencing machineries of plants and insect vectors to allow virus replication and infection in the host organism. The *NS4* gene encodes the disease-typical protein that accumulates in infected tissues (Lu et al., 2017, 2019). In the susceptible control BB50, the amplification of RHBV genes was significant since it lacks a mechanism that prevents virus replication. A similar scenario occurred in the edited lines, although to a lesser extent, where the virus replicated due to the malfunction in the expression of the AGO4 gene, resulting in an increase in the amplification of NP, *NS3*, and *NS4*. This was completely different in FD2000, where the amplification of these genes was not perceptible or was absent.

The expression patterns of a gene are key to elucidating when it is related to a phenotypic characteristic. In the WT, the RHBV-resistant variety FD2000, it was demonstrated how the AGO4 gene increases its expression levels when the plants are under the effects of mechanical stress by the insect *T. orizicolus* and infected with the virus, but as time increases, overexpression is only increased or maintained in RHBV-infected plants by virus activity. A completely different scenario was found in the plants of the susceptible variety BB50, where from day 4, due to both mechanical stress and viral stress, the expression of the gene decreased, reinforcing why this variety is completely vulnerable to RHBV. By generating a knockdown of the AGO4 gene, we managed to reduce the expression in the edited lines infected with the virus and with mechanical stress. When comparing the expression levels of AGO4 and the amplification of the NP, *NS3*, and *NS4* genes, it was evident how the decrease in the expression of AGO4 only occurred in the edited plants where the virus genes were amplified and the symptoms of RHBV appeared, which demonstrates the relationship of the AGO4 gene with the resistance response to RHBV in the *O. sativa* variety FD2000. We propose that future studies analyze the interaction between the expression of the AGO4 and *NS3* genes in control and edited plants and the RNA silencing mechanism of resistant plants in comparison to the viral mechanism of suppression of RNA silencing mechanisms of the host. We hypothesize that it is a game of which manages to suppress the other first, resulting in a negative regulation of gene expression.

The function of a protein is defined by its tertiary structure and the domains that shape it. The presence of the N-terminal functional domains, PAZ, MID, and PIWI (the C-terminal is located within the PIWI domain), and linker segments (L1 and L2) in the AGO4 protein were determined. The PAZ and MID domains perform the function of anchoring the sRNA. The PIWI domain is the catalytic region of the protein, responsible for cleavage of the target RNA. By performing multiple alignments of the amino acid sequences of the PIWI domains of different argonaute proteins from other species, cataloged with the sRNA silencing function together with the sequence of the WT, we found that the PIWI domain was highly conserved. Similarly, within the PIWI domain, we found the presence of the DDH catalytic triad (Asp-Asp-His), which is reported to be the functional unit that performs RNA cutting in the PIWI domain (Mallory and Vaucheret, 2010; Meng et al., 2013; Rodriguez-Leal et al., 2016). Therefore, the presence of the DDH catalytic triad and the similarity of the PIWI domain of the AGO4 protein in rice explains how it is involved in mediating a response in the silencing of the viral RNA.

The structure of the AGO4 protein and its mutant version was modeled to elucidate how much the tertiary structure changes due to the mutation. The WT protein consists of 911 residues. Because the mutation was made in the last exon, residues 885 to 911 were lost, for a total of 27 residues deleted. This generated a loss of the last stretch of the PIWI domain and the C-terminal. It did not change the structure of the protein to a large extent, but previous studies indicate that the residues located in the C-terminal are necessary for the correct positioning of the RNA to be cleaved. By eliminating them, the cut in the sRNA would not occur or would be an inefficient cut (Mallory and Vaucheret, 2010).

These findings on argonaute-mediated antiviral defense, particularly involving AGO4 in *Oryza sativa*, can be applied to crop improvement programs and resistance engineering in several ways. For rice, strategies could focus on enhancing AGO4 expression or function to strengthen resistance to RHBV, as seen in the resistant variety FD2000. The role of AGO proteins in binding small RNAs (e.g., vsiRNA) and silencing viral RNA can be harnessed to develop genetically engineered lines with overexpressed or optimized AGO proteins. Additionally, the understanding of viral counter-defense mechanisms, such as suppression of RNA silencing (e.g., *NS3* protein in RHBV or HC-Pro in TuMV), could inform the design of plants that either prevent viral proteins from interacting with AGO proteins or maintain AGO protein stability under viral stress.

For other susceptible crops, such as those vulnerable to economically significant viruses, the application of CRISPR/Cas9 technology could target AGO-related pathways, optimizing their role in RNA silencing without compromising critical developmental processes, as demonstrated in rice mutation studies. This approach, combined with structural insights into functional AGO domains such as PIWI, could ensure both enhanced resistance and minimal off-target effects on plant growth or reproduction.

In conclusion, this study demonstrates how a mutation in the PIWI domain of the AGO4 gene created by CRISPR/Cas9 editing was used as a tool to reveal the functional relationship of the gene in resistance to RHBV in the resistant rice variety FD2000.

## Data Availability

The original contributions presented in the study are included in the article/**Supplementary Material**. Further inquiries can be directed to the corresponding authors.
