# Supplementary material for: Revealing the role of the AGO4 gene against rice hoja blanca virus: from transformation to protein structure
Source: Front Plant Sci. 2025 Mar 28;16:1517321. doi: 10.3389/fpls.2025.1517321 (PMC11985844; doi:10.3389/fpls.2025.1517321)
Supplement: Supplementary file 1 [file DataSheet1.pdf]

Supplementary Material

Table S1. List of Off targets sites identified through CRISPR-P v2.0 web platform.

Target 1 (AGO4-1) : CCTACCGTTCTGAGCTTTCAAG / CTTGAAAGCTCAGAACGGTAGG

Sequence	Off-score	MMs	Locus	Gene	Region
ACTTGAAAGCTCAGAAACCTTGG	0.090	4MMs	3:-30276969		Intergenic
GCTTGCTTGCTCAGAACTGTTGG	0.057	4MMs	11:+21179270	OS11G0568300	intron
GCTCGCAAGCTCATAACCGTTGG	0.037	4MMs	5:-22618634	OS05G0460200	exon
CCTTGAAAGCTCAGATCAGCAGG	0.000	4MMs	7:+24769225		Intergenic

Target 2 (AGO4-2) : CCAAGGAGGTCATACGTCTGTC/ GACAGACGTATGACCTCCTTGG

Sequence	Off-score	MMs	Locus	Gene	Region
AGACAGACGAATGACATCATGGG	0.367	4MMs	3:+26182480		Intergenic
CGAAAGACGTATATCCTGCTCGG	0.055	4MMs	12:-19162243	OS12G0502800	exon

Table S2. Transformation efficiency.

Target	# of transformations	# total plants/transformation	Dead	# of negative plants	# positive plants
Ago 1	3	40	1	39	0
Ago 2	5	82	4	58	20

Ago 1:

- total plants/transformation = 40
- positive plants = 0
- $Efficiency = \left(\frac{0}{40}\right) \times 100 = 0\ \%$

Ago 2:

- total plants/transformation = 82
- positive plants = 20
- $Efficiency = \left(\frac{20}{82}\right) \times 100 \approx 24.39\ \%$

Transformation Efficiency Results:

- **Ago 1**: 0%
- **Ago 2**: Approximately 24.39%

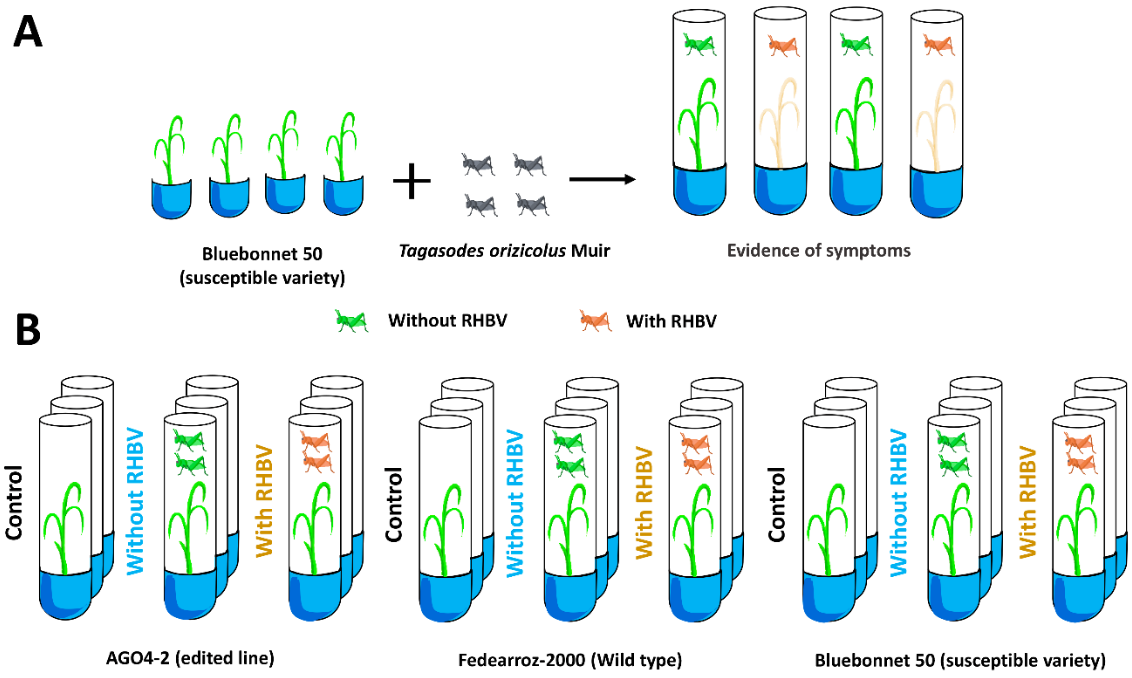

Figure S1. Graphic diagram of infestation. The susceptible variety BB50 was used as a susceptible control to establish which insects were vectors and which were healthy before proceeding with the infestation of the experimental lines.

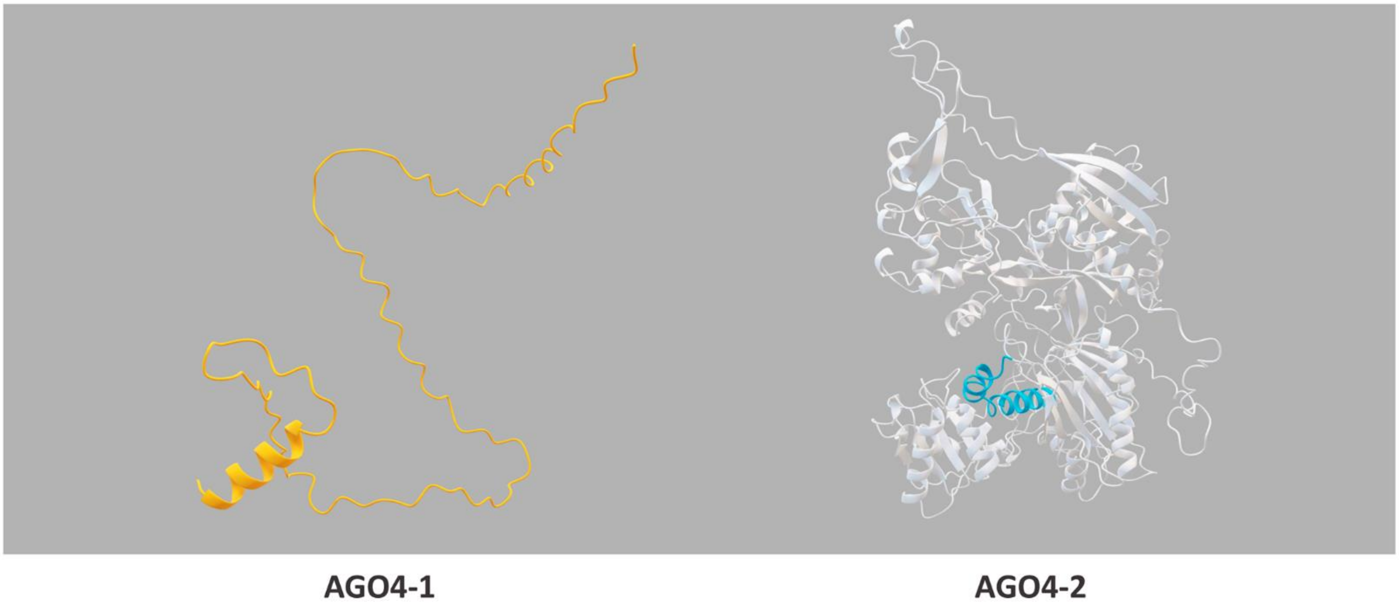

Figure S2. Modeling of the AGO4-1 and AGO4-2 editing sites.

Table S3. The primers used in this study.

ID	Sequence	Tm
eEF-1a-Fw	TTTCACTCTTGGTGTGAAGCAGAT	56.7
eEF-1a-Rv	GACTTCCTTCACGATTTCATCGTAA	55
GAPDH-Fw	AAGCCAGCATCCTATGATCAGATT	56.2
GAPDH-Rv	CGTAACCCAGAATACCCTTGAGTTT	56.7
AGO4-1-Fw	GCCTGTTTACAATTGGTGCTC	58
AGO4-1-Rv	TCTCTTCCTGTCACTACCTCC	58.5
NS3-FW2	AACCAAACAAGTCAGAGGCAG	55
NS3-RV2	AGTGCGATATGGATGCTCTCT	55
NS4-FW3	TTGGCTCTACCTTTGATTCC	55
NS4-RV3	ACTCACTTCACCATTGACTC	55
NCP-FW1	CTAGAGAGGTCCAGAAAG	55
NCP-RV1	CTCAAGGTAGAGAAGAAGA	55
